# The role of effective factors on suicidal tendency of women in Turkey

**DOI:** 10.3389/fpubh.2023.1332937

**Published:** 2024-01-10

**Authors:** Şerife Kılıçarslan, Sefa Çelik, Abdullah Y. Güngör, Ömer Alkan

**Affiliations:** ^1^Oltu Faculty of Humanities and Social Sciences, Department of Finance and Banking, Ataturk University, Erzurum, Türkiye; ^2^Faculty of Economics and Administrative Sciences, Department of Business Administration, Ataturk University, Erzurum, Türkiye; ^3^Oltu Faculty of Humanities and Social Sciences, Department of Business Administration, Ataturk University, Erzurum, Türkiye; ^4^Faculty of Economics and Administrative Sciences, Department of Econometrics, Ataturk University, Erzurum, Türkiye; ^5^Master Araştırma Eğitim ve Danışmanlık Hizmetleri Ltd. Şti., Erzurum, Türkiye

**Keywords:** suicide attempts, suicidal tendency, suicide risk factors, intimate partner violence, binary logistic regression, Turkey

## Abstract

**Background/Aim:**

This study aims to identify the variables that influence the suicidal tendency of women who are married, have had a relationship or are currently in a relationship in Turkey.

**Methods:**

This study uses cross-sectional data from the 2014 Hacettepe University Institute of Population Studies National Research on Domestic Violence Against Women in Turkey. Data from 6,458 women between the ages of 15 and 49 were analyzed in this dataset. Binary logistic regression was used to determine the factors influencing women’s suicidal tendencies.

**Results:**

Based on the analysis’s findings, age, education level, health status, number of children, the sector in which the spouse/partner works, the drinking status of the spouse/partner, the situation where the spouse/partner fights with another man in a way that involves physical violence, the cheating status of the spouse/partner, the controlling behaviour of the spouse/partner, exposure to various types of violence by both the spouse/partner and someone other than the partner, and the household income level variables were found to be associated with the suicidal tendency of women.

**Conclusion:**

Prioritizing women who are, in particular, between the ages of 15 and 24, live in the south of Turkey, have a high school education, are in poor health, are childless, have low household incomes, live with an unemployed spouse or partner, and are exposed to various forms of violence from their partner or other sources can be achieved more effective results in reducing and preventing women’s suicidal behaviors.

## 1 Introduction

Suicide is the deliberate taking of one’s own life, which ends in death. If it does not result in death, it is called a suicide attempt (1). Suicide, which begins with mental and behavioral planning, is a complex behavior that culminates in fatal or nonfatal attempts (2). Suicidal behavior, thought, planning, and attempts are serious, avoidable public health issues that significantly increase morbidity and mortality and add to the global disease burden (3, 4). Although the majority of suicide attempts do not end in death, they pose a risk of severe injury and increase the likelihood of a second attempt (5). According to studies, the risk of suicide/death in suicide attempters increases 30–40 times compared to the general population (6).

Suicide, an important global public health problem, has become a growing social problem as interaction and communication complexity in existing societies has increased (7). According to the report of the World Health Organization (WHO) in 2021, more than 700,000 people end their lives by suicide every year. This shows that one person commits suicide every 40 s. 1.3% of all deaths in the world occur by suicide. In addition, while suicide ranks seventeenth among the causes of death, it has risen to fourth place among individuals between the ages of 15–29 (8).

The rate of suicide attempts, which has reached a high level, has demonstrated that this behavior should be addressed appropriately around the world. Studies have determined that suicide events that are not intervened with the appropriate methods and promptly increase the likelihood of recurrence and the risk of achieving the goal of dying (9). Suicide-inducing behaviors are not random occurrences. Events that develop over time and hurt a person’s mental health compel that person to develop suicidal tendencies over time. Although the units where individuals at risk of suicidal behavior are treated are psychiatry services, it is known that there are individuals who attempt suicide even during the treatment process (10). It is important to classify and understand the factors that cause suicide correctly and to develop effective strategies to prevent and reduce it (11). Suicidal tendency is prevalent in all social groups and classes (2). Considering the studies examining suicide cases around the world, it was concluded that the factors triggering suicide for women and men may differ (12–16).

In the literature, there are remarkable studies on suicide and gender (17–28). According to some of these studies, there was no difference in self-harm between the sexes (17, 19, 23). Studies also report that women have a higher rate at this point (20, 25). This difference can be explained by the fact that both species view events and situations differently. For instance, while men can more easily control their emotions, women tend to act more emotionally (29, 30). This emotional structure can make it difficult for women to cope with events. These difficult times, which cannot be overcome, can manifest in women as self-harm or suicide. The use of less painful suicide methods (medication, wrist cutting, etc.) by women may also be indicative of their emotional characteristics (31). In addition, the fact that women are exposed to more abuse than men is shown as one of the reasons why suicidal ideation is more common in women (7).

Examining studies of women’s suicidal behaviors and causes reveals that women’s greater willingness to accept assistance and treatment with alcohol and drugs (32) and having small children (28) reduce the risk of suicidal thoughts and behavior. It is also seen that the suicide risk of a never-married woman is lower than that of a married woman and a man (33). In another study, hopelessness was found to be closely associated with suicidal thoughts in women (34). In addition, studies suggest that obesity (35), depressive processes (36, 37), alcoholism (38), and, albeit infrequently, advanced age (39) and fatigue (40) induce suicide in women.

Suicidal ideation refers to *the act of thinking about or a state of preoccupation with taking one’s own life; the act of considering or planning suicide* (Merriam-Webster (41)). Recent studies have revealed that women have higher suicidal ideation than men (42–48). These studies can be examined as suicidal ideation in adolescent, pregnant, and working women. Adolescent women have higher suicidal ideation than adolescent men (49–52). Factors that increase suicidal ideation in adolescent women are menarche, irregular menstrual cycle, overweight (53), and public stigma (54). The pregnancy period in women is expressed as a period of high risk for suicidal ideation (55). Factors that increase suicidal ideation during this period are unplanned pregnancy, poor social support, common mental disorders (56), intimate partner violence (57), depressive symptoms (58), anxiety, lower level of education, age, and an unemployed professional status (59, 60). Although an unemployed professional status increases suicidal ideation in women, work and family stresses in working women raise suicidal ideation as well (61).

Apart from studies focused on adolescent, pregnant, and working women, some previous studies have investigated suicidal ideation in women in general (46, 48, 62, 63). According to the findings of these studies, factors that affect suicidal ideation in women are loneliness (63), alcohol consumption (62), age, depression, stress, social support (48), bipolar disorder, depressive symptoms, eating disorder, interpersonal problems, posttraumatic stress disorder, previous abortion, being a victim of dating violence (46).

Many studies have found that victims of domestic violence have higher rates of suicidal ideation, similar to victims of dating violence (11). Researchers and human rights advocates have recently paid considerable attention to domestic violence as a cause of suicide attempts among women (64). Partner violence was identified as one of the most consistent risk factors for female suicide attempts in a separate study (65). When the starting point of the causes of suicide in general is examined, it is determined that there is violence in the family or close environment experienced during childhood and/or adolescence (66). Violence can also affect the physical and mental health of children and/or adolescents, making them vulnerable to issues like social isolation, alcoholism, anxiety disorders, post-traumatic stress disorder, depression, and suicidal ideation (67, 68).

When suicide rates are compared on a regional basis as Africa, the Americas, the Eastern Mediterranean, Europe, Southeast Asia and the Western Pacific, Europe has the second highest rate. In Turkey, located in this region, the total number of suicides committed in 2019 was 2,003. Five hundred seventeen of them are women. While the female suicide rate in Turkey was 1.8 per 100,000 in 2000, this rate dropped to 1.2 by 2011. Despite this significant progress, by 2019, the relevant rate remained at 1.2 (8). Although men account for the majority of fatal suicides in Turkey, this situation may vary between regions. In Turkey, the rate of female suicide is observed to increase from west to east (69). For instance, it has been determined that women commit suicide at a higher rate than men in Southeastern Anatolia (70). In another study, one-year suicide cases were examined in a hospital, and it was determined that 64.18% of 416 patients were women (71). In another study conducted in Adıyaman, it was determined that 71.9% of patients who committed suicide within a given period were women (72).

The majority of suicides (79%) occur in low- and middle-income countries (2). As a result of Islam’s prohibition on suicide, the rate of suicide is relatively low in Islamic countries, but it is on the rise in these regions (73). For this reason, this study aims to determine the factors affecting the suicidal tendency of women who are married, have had a relationship or are still in a relationship in Turkey. To examine the effects of sociodemographic and economic factors of the spouse/partner on the suicidal tendencies of women, only married women who had been in a relationship or were currently in a relationship were included in the study. The complex interaction of numerous factors causes suicide. Despite the difficulty of predicting suicide, certain sociodemographic risk factors can be identified. These factors will also be examined from a regional and demographic standpoint. According to the literature review, although there are regional or provincial suicide studies in Turkey, there is no study that examines Turkey as a whole and explains regional differences. This study will add a unique value to the literature as a source for analysing Turkey. Survey data obtained from the TURKSTAT research survey were used in the study. These data are utilized because they reflect the country as a whole and because the study allows international comparisons and sheds light on national needs.

## 2 Methods

### 2.1 Data source

In this study, the cross-sectional data of the National research on domestic violence against women in Turkey conducted by Hacettepe University Institute of Population Studies in 2014 were used. The most recent National research on domestic violence against women in Turkey data shared by Hacettepe University Institute of Population Studies is that of the year 2014. This research’s main strength and advantage is very significant, and unfortunately, the actual topic is always suicidality. A particularly important and neglected topic is women’s mental health. The sample size is more than enough, although the data is from 10 years ago.

The National research on domestic violence against women in Turkey is one of the most comprehensive studies conducted nationwide to understand the dimension, content, causes, effects and risk factors of domestic violence experienced by women in Turkey. It was initially displayed in 2008 to ascertain the various facets and causes of violence against women as well as to fulfil the need for data collection on this matter. In terms of illustrating the evolution of domestic violence against women since the 2008 research, the National Research on Domestic Violence Against Women in Turkey conducted in 2014 is noteworthy (74).

The research questionnaire was designed by taking the questionnaires used by WHO’s “Multi-country Study on Women’s Health and Domestic Violence against Women” into account (75). New questions have been added to the questionnaire per the country’s needs, focusing on legal regulations (74).

Within the scope of the research on violence, Turkey was divided into 30 strata to provide estimates at the national, urban/rural, 12 regionals, and five regional levels. Except for the Istanbul region, one of the 12 regions, the ratio between urban and rural populations in the remaining areas is approximately 75 to 25%. About 5 per cent of the households in Istanbul were chosen from rural areas. In the research, settlements with a population of 10,000 or more constitute urban, and settlements with less than 10,000 constitute rural strata. The research sample is cluster sampling (74).

April 8, 2014, marked the beginning of the study’s 2014 field application, which ended on July 11, 2014 (74). The research team distributed the questionnaires for the study on domestic violence against women in Turkey. At every stage of the study, the ethical guidelines established by the World Health Organization were adhered to, and steps were taken to guarantee the security of the research team and the women questioned. Consent was secured from each respondent before the interview, and interviewees signed the questionnaire attesting to this fact. The researchers were aware of the subject’s sensitivity before, during, and after the interview because they had received training on the Code of Ethics and Safety. If there were multiple women in the family between the ages of 15 and 59, the interviews were done with one randomly chosen woman from each household. This was done to avoid asking the same questions to multiple women in the household. The interviews were done in a confidential location thanks to the research teams’ exceptional attention to detail. Additionally, instruction on interview confidentiality was given to each interviewee. Additionally, respondents were notified that their responses would be kept private during the approval and notification process (74).

In the 2014 survey, 7,462 women completed the questionnaire and were interviewed in person; the study’s rejection rate was 4.4%. In interviews with women, the response rate was 83.3% (74). These records were supplemented with women’s weights determined in compliance with the study’s sample design (74).

Household surveys (which asked about the number of people living in the home, the number of rooms, and welfare indicators) and women’s questionnaires (which asked about sociodemographic information and other details about the woman and her spouse/partner) were used in the Domestic Violence Against Women Research in Turkey. Two different Excel files containing these data were sent. Two different Excel files containing these data were sent. After the merger, 7,070 women’s data were processed, and the analysis did not include 392 women whose household information could not be collected.

When the data of 612 women who had never been in a relationship at the time of the survey were removed, the number of units was determined to be 6,458 in the study because the suicidal inclination of married, single, or involved women was studied.

### 2.2 Outcome variable

In the Research on Domestic Violence Against Women in Turkey, women were asked the following questions about suicidal tendencies. “Have you ever contemplated suicide?” and “Have you ever attempted suicide?” The suicidal tendency measured by these questions served as the dependent variable. If the women participating in the study answered yes to one or more of the conditions mentioned, they had a suicidal tendency. If they did not experience any of them, they did not have a suicidal tendency. As a result, the dependent variable of the study is the suicidal tendency status of the women who received the code 1 if women have a suicidal tendency and 0 otherwise.

### 2.3 Independent variables

In this study, which examines the suicidal tendency of women, sociodemographic, economic, and domestic violence questions asked of survey participants were analyzed, and variables predicted to be effective were included in the model.

Variables related to sociodemographic and economic characteristics of women are as follows: region (west, south, central, north, east), age (15–24, 25–34, 35–44, 45–54, 55 and over), education level (illiterate, primary school, secondary school, high school, university), state of health (excellent/good, poor/very poor, moderate), number of children (no children, one child, two or more), spouse/partner’s employment status (unemployed, public, private), spouse/partner’s drinking status (no, yes), the situation where the spouse/partner fights with another man in a way that involves physical violence (no, yes), spouse/partner’s cheating status (no, yes), if the spouse/partner prevents the woman from meeting with her friends (no, yes), the situation where the spouse/partner interferes with the clothes (no, yes), if the spouse/partner interferes with the woman’s use of social media (no, yes), exposure to economic violence by spouse/partner (no, yes), the state of being exposed to emotional violence by the spouse/partner (no, yes), being exposed to physical violence by the spouse/partner (no, yes), sexual violence by the spouse/partner (no, yes), exposure to physical violence by someone other than a spouse/partner (no, yes), exposure to sexual violence by someone other than spouse/partner (no, yes), exposure to emotional violence by someone other than spouse/partner (no, yes), exposure to economic violence by someone other than spouse/partner (no, yes) and household income level (1st Income level (min), 2nd Income level, 3rd Income level, 4th Income level (max)).

This analysis only takes into account categorical variables with two-state or ordinal scales. To observe the impact of the categories of all the variables to be included in the binary logistic regression model, ordinal and nominal variables were defined as dummy variables (76).

### 2.4 Statistical analysis

Survey statistics in Stata 15 (Stata Corporation) were used to account for the complex sampling design and weights. Weighted analysis was performed (77). First, the study’s participant women’s frequencies and percentages were calculated based on how often they were exposed to sexual violence by their partners or spouses. The association between sexual assault and independent variables was investigated using the chi-square independence test. The categories from which any observed differences originate are also revealed by the Pearson chi-square (*χ*^2^), in addition to the importance of the reported differences (78). Then, suicide risk variables were found using binary logistic regression analysis.

## 3 Results

### 3.1 Characteristics of study participants

The frequency and percentages of the independent variables connected to the proposed model will be interpreted in this section. The variables influencing women’s exposure to sexual violence are shown in Table 1, along with the Chi-Square test findings.

**Table 1:** Factors affecting suicide tendency of women and chi-square test statistics.

Variables		Suicide tendency		*n* (%)	*χ* ^2^	*P*
		No	Yes			
Region	West	1712 (32.5)	425 (35.6)	2,137 (33.1)	10.655	0.031
	South	411 (7.8)	113 (9.5)	524 (8.1)		
	Central	1,112 (21.1)	239 (20.0)	1,351 (20.9)		
	North	773 (14.7)	149 (12.5)	922 (14.3)		
	East	1,255 (23.8)	269 (22.5)	1,524 (23.6)		
Age	15–24	613 (11.8)	180 (15.1)	803 (12.4)	26.588	0.000
	25–34	1,626 (30.9)	359 (30.0)	1985 (30.7)		
	35–44	1,398 (26.6)	352 (29.5)	1750 (27.1)		
	45–54	1,207 (22.9)	205 (17.2)	1,412 (21.9)		
	55+	409 (7.8)	99 (8.3)	508 (7.9)		
Education	Illiterate	1,017 (19.3)	224 (18.7)	1,241 (19.2)	24.232	0.000
	Primary school	2,387 (45.4)	520 (43.5)	2,907 (45.0)		
	Secondary school	669 (12.7)	210 (17.6)	879 (13.6)		
	High school	748 (14.2)	168 (14.1)	916 (14.2)		
	University	442 (8.4)	73 (6.1)	515 (8.0)		
Health State	Excellent/good	2,607 (49.5)	350 (29.3)	2,957 (45.8)	206.212	0.000
	Moderate	2079 (39.5)	574 (48.0)	2,653 (41.1)		
	Poor/Very poor	577 (11.0)	271 (22.7)	848 (13.1)		
Number of Children	None	696 (13.2)	201 (16.8)	897 (13.9)	11.841	0.003
	One	834 (15.8)	166 (13.9)	1,000 (15.5)		
	Two or more	3,733 (70.9)	828 (69.3)	4,561 (70.6)		
Sector in which the spouse/partner works	Unemployed	963 (18.3)	267 (22.4)	1,230 (19.1)	24.063	0.000
	Public	719 (13.7)	109 (9.2)	828 (12.8)		
	Private	3,571 (68.0)	815 (68.4)	4,386 (68.1)		
Drinking status of the spouse/partner	No	4,327 (82.2)	839 (70.2)	5,166 (80.0)	87.720	0.000
	Yes	936 (17.8)	356 (29.8)	1,292 (20.0)		
The situation where the spouse/partner fights with another man in a way that involves physical violence	No	4,843 (92.0)	960 (80.3)	5,803 (89.9)	145.903	0.000
	Yes	420 (8.0)	235 (19.7)	655 (10.1)		
Spouse/partner’s cheating status	No	4,876 (92.6)	910 (76.2)	5,786 (89.6)	284.262	0.000
	Yes	387 (7.4)	285 (23.8)	672 (10.4)		
If the spouse/partner prevents the woman from meeting with her friends	No	4,676 (88.8)	861 (72.1)	5,537 (85.7)	224.699	0.000
	Yes	587 (11.2)	334 (27.9)	921 (14.3)		
The situation where the spouse/partner interferes with the clothing of the woman	No	3,610 (68.6)	634 (53.1)	4,244 (65.7)	104.356	0.000
	Yes	1,653 (31.4)	561 (46.9)	2,214 (34.3)		
If the spouse/partner interferes with the woman’s use of social media	No	4,391 (83.4)	854 (71.5)	5,245 (81.2)	91.426	0.000
	Yes	872 (16.6)	341 (28.5)	1,213 (18.8)		
Exposure to economic violence by spouse/partner	No	3,985 (76.7)	637 (54.0)	4,622 (72.5)	247.225	0.000
	Yes	1,212 (23.3)	542 (46.0)	1754 (27.5)		
Exposure to emotional violence by the spouse/partner	No	3,402 (64.6)	393 (32.9)	3,795 (58.8)	405.213	0.000
	Yes	1861 (35.4)	802 (67.1)	2,663 (41.2)		
Exposure to physical violence by the spouse/partner	No	3,813 (72.4)	516 (43.2)	4,329 (67.0)	377.537	0.000
	Yes	1,450 (27.6)	679 (56.8)	2,129 (33.0)		
exposure to sexual violence by the spouse/partner	No	4,882 (92.8)	873 (73.1)	5,755 (89.1)	389.864	0.000
	Yes	381 (7.2)	322 (26.9)	703 (10.9)		
Exposure to sexual violence by someone other than a spouse/partner	No	4,718 (89.6)	892 (74.6)	5,610 (86.9)	192.107	0.000
	Yes	545 (10.4)	303 (25.4)	848 (13.1)		
Exposure to emotional violence by someone other than a spouse/partner	No	5,035 (95.7)	1,025 (85.8)	6,060 (93.8)	164.843	0.000
	Yes	228 (4.3)	170 (14.2)	398 (6.2)		
Exposure to physical violence by someone other than a spouse/partner	No	4,379 (83.2)	758 (63.4)	5,137 (79.5)	233.998	0.000
	Yes	884 (16.8)	437 (36.6)	1,321 (20.5)		
Exposure to economic violence by someone other than a spouse/partner	No	3,302 (62.7)	596 (49.9)	3,898 (60.4)	67.370	0.000
	Yes	1961 (37.3)	599 (50.1)	2,560 (39.6)		
Household income level	1st Income level (min)	1,281 (24.3)	357 (29.9)	1,638 (25.4)	26.209	0.000
	2nd Income level	1,305 (24.8)	286 (23.9)	1,591 (24.6)		
	3rd Income level	1,310 (24.9)	312 (26.1)	1,622 (25.1)		
	4th Income level (max)	1,367 (26.0)	240 (20.1)	1,607 (24.9)		

Table 1’s chi-square independence test findings indicate that there is a substantial correlation between all parameters and women’s suicidality.

According to Table 2, 33.1% of the survey sample is comprised of women from the western region, while 8.1% are from the southern region. Women in the 25–34 age group constitute 30.7% of the study group, and women aged 55 and over make up 7.9% of the study group. Primary school graduate women constitute 45% of the sample and university graduates 8%. Women with excellent/good health state constitute 45.8% of the sample, and those with poor/very poor health constitute 13.1%. Women with no children constitute 13.9% of the sample, while those with two or more children constitute 70.6%.

**Table 2:** Estimated model results of factors affecting women’s suicide tendency.

Variables		*β*	Standard deviation	*P*	VIF
Constant term		−1.562	0.279	0.000	
Region (reference: south)					
	West	−0.038	0.143	0.785	3.41
	Central	−0.327	0.156	0.036	2.85
	North	−0.154	0.164	0.347	2.40
	East	−0.310	0.159	0.051	3.19
Age (reference: 45–54)					
	15–24	0.452	0.191	0.018	2.12
	25–34	0.343	0.135	0.011	2.05
	35–44	0.480	0.125	0.000	1.75
	55+	0.360	0.172	0.037	1.28
Education (reference: high school)					
	Illiterate	−0.414	0.164	0.012	2.62
	Primary School	−0.348	0.135	0.010	2.99
	Secondary School	−0.125	0.157	0.428	1.91
	University	−0.149	0.203	0.461	1.75
Health State (reference: poor/very poor)					
	Excellent/good	−1.059	0.128	0.000	2.85
	Moderate	−0.398	0.114	0.001	2.53
Number of Children (reference: none)					
	One	−0.354	0.161	0.029	2.06
	Two or more	−0.363	0.152	0.018	2.77
Sector in which the spouse/partner works (reference: unemployed)					
	Public	−0.413	0.169	0.015	1.65
	Private	−0.270	0.110	0.015	1.66
Drinking status of the spouse/partner (reference: no)					
	Yes	0.302	0.100	0.003	1.16
The situation where the spouse/partner fights with another man in a way that involves physical violence (reference: no)					
	Yes	0.224	0.133	0.093	1.13
Spouse/partner’s cheating status (reference: no)					
	Yes	0.359	0.122	0.003	1.18
If the spouse/partner prevents the woman from meeting with her friends (reference: no)					
	Yes	0.219	0.120	0.069	1.25
The situation where the spouse/partner interferes with the clothing of the woman (reference: no)					
	Yes	0.269	0.092	0.003	1.22
If the spouse/partner interferes with the Woman’s use of social media (reference: no)					
	Yes	0.186	0.110	0.092	1.26
Exposure to economic violence by spouse/partner (reference: no)					
	Yes	0.346	0.095	0.000	1.22
Exposure to emotional violence by spouse/partner (reference: no)					
	Yes	0.449	0.105	0.000	1.50
Exposure to physical violence by spouse/partner (reference: no)					
	Yes	0.415	0.104	0.000	1.56
Exposure to sexual violence by spouse/partner (reference: no)					
	Yes	0.635	0.122	0.000	1.30
Exposure to physical violence by someone other than a spouse/partner (reference: no)					
	Yes	0.271	0.118	0.022	1.23
Exposure to sexual violence by someone other than a spouse/partner (reference: no)					
	Yes	0.712	0.142	0.000	1.11
Exposure to emotional violence by someone other than a spouse/partner (reference: no)					
	Yes	0.245	0.106	0.021	1.27
Exposure to economic violence by someone other than a spouse/partner (reference: no)					
	Yes	0.212	0.091	0.020	1.16
Household income level [reference: 4th income level (max)]					
	1st income level (min)	0.222	0.129	0.085	1.76
	2nd income level	−0.021	0.123	0.861	1.56
	3rd income level	0.259	0.122	0.033	1.54
Classification success				0.837	
Pseudo *R*^2^				0.177	
Cox Snell/ML *R*^2^				0.156	
AIC				5079.05	
BIC				5322.35	
Log-likelihood				−2503.53	

Those whose spouses/partners do not work account for 19.1% of the sample, while those whose spouses/partners work in the private sector account for 68.1%. 20% of the sample consists of women whose spouses/partners drink, while 10.4% of the sample consists of women whose spouses/partners have cheated on them. 14.3% of the sample consists of women whose spouses/partners prevent them from meeting with friends. Women whose spouse/partner interferes with the clothes accounted for 34.3% of the sample. Women whose spouse/partner interferes with the use of social media constitute 18.8% of the sample. Women exposed to economic, emotional, physical and sexual violence by their spouses/partners constitute 27.5, 41.2, 33 and 10.9% of the sample, respectively. Women who have been exposed to physical, sexual, emotional and economic violence by someone other than their spouses/partners constitute 13.1, 6.2, 20.5 and 39.6% of the sample, respectively.

### 3.2 Multivariate analyses

The study employed the binary logistic regression model to identify the variables influencing the suicidal tendencies of the female participants. Table 2 presents the estimated model findings.

According to the data presented in Table 2, the region, age, education level, health status, number of children, the sector in which the spouse/partner works, the drinking status of the spouse/partner, the spouse/partner’s fight with another man involving physical violence, the spouse/partner’s cheating status, the spouse/partner’s obstruction of friend meetings, the interference with clothing and social media use, the spouse/partner’s economic, emotional, physical and sexual violence, exposure to physical, sexual, emotional and economic violence of someone other than spouse/partner and household income level variables are found to be significant.

### 3.3 Marginal effects

Table 3 shows the marginal effects of factors affecting women’s suicidality. Additionally, multicollinearity between the model’s independent variables was examined. Those with variance inflation factor (VIF) values of 5 and above are thought to cause moderate multicollinearity, and those with values of 10 and above cause a high degree of multicollinearity (79). According to the VIF results presented in Table 3, there is no variable that causes the multicollinearity problem between the variables.

**Table 3:** Estimated marginal effect values of factors affecting women’s suicide tendency.

Variables		Marginal effects	Std. Error
Region (reference: south)			
	West	−0.031	0.112
	Central	−0.264^b^	0.125
	North	−0.123	0.131
	East	−0.250^c^	0.127
Age (reference: 45–54)			
	15–24	0.369^b^	0.154
	25–34	0.283^b^	0.111
	35–44	0.391^a^	0.103
	55+	0.296^b^	0.140
Education (reference: high school)			
	Illiterate	−0.330^b^	0.131
	Primary School	−0.277^b^	0.106
	Secondary School	−0.097	0.122
	University	−0.117	0.159
Health State (reference: poor/very poor)			
	Excellent/good	−0.840^a^	0.099
	Moderate	−0.298^a^	0.083
Number of Children (reference: no)			
	One	−0.279^b^	0.126
	Two or more	−0.286^b^	0.117
Sector in which the spouse/partner works (reference: unemployed)			
	Public	−0.331^b^	0.137
	Private	−0.214^b^	0.086
Drinking status of the spouse/partner (reference: no)			
	Yes	0.240^a^	0.079
The situation where the spouse/partner fights with another man in a way that involves physical violence (reference: no)			
	Yes	0.178^c^	0.104
Spouse/partner’s cheating status (reference: no)			
	Yes	0.283^a^	0.094
If the spouse/partner prevents the woman from meeting with her friends (reference: no)			
	Yes	0.174^c^	0.094
The situation where the spouse/partner interferes with the clothing of the woman (reference: no)			
	Yes	0.216^a^	0.073
If the spouse/partner interferes with the Woman’s use of social media (reference: no)			
	Yes	0.148^c^	0.087
Exposure to economic violence by spouse/partner (reference: no)			
	Yes	0.276^a^	0.075
Exposure to emotional violence by spouse/partner (reference: no)			
	Yes	0.363^a^	0.085
Exposure to physical violence by spouse/partner (reference: no)			
	Yes	0.333a	0.083
Exposure to sexual violence by spouse/partner (reference: no)			
	Yes	0.493^a^	0.090
Exposure to physical violence by someone other than a spouse/partner (reference: no)			
	Yes	0.215^b^	0.092
Exposure to sexual violence by someone other than a spouse/partner (reference: no)			
	Yes	0.545^a^	0.103
Exposure to emotional violence by someone other than a spouse/partner (reference: no)			
	Yes	0.196^b^	0.084
Exposure to economic violence by someone other than a spouse/partner (reference: no)			
	Yes	0.170^b^	0.073
Household income level (reference: 4th Income level (max))			
	1st income level (min)	0.179^c^	0.104
	2nd income level	−0.017	0.101
	3rd income level	0.208^b^	0.098

^a^*p* < 0.01; ^b^*p* < 0.05; ^c^*p* < 0.10.

According to the binary logistic regression model given in Table 3, women residing in the middle region are 26.4% less likely to be suicidal than women residing in the south region, all other variables being constant. Similarly, women residing in the eastern region are 25% less likely to be suicidal than women residing in the southern region. The suicidal tendency of 35-44-year-old women is 39.1% higher than that of 45-54-year-old women. Similarly, women aged 15–24 have a suicidal tendency that is 36.9% greater than women aged 45–54. When compared to women aged 45–54, the suicidal tendencies of women over 55 and women aged 25–34 are 29.6 and 28.3% higher, respectively, than those of women aged 45–54. Considering the education level, the suicide tendency of illiterate and primary school graduates is 33 and 27.7% less, respectively, than high school graduates. Women with excellent or good health are 84% less suicidal than women with poor or very poor health. Women with one child have a suicidal tendency that is 27.9% lower than women with no children. Likewise, women with two or more children are 28.6% less likely to commit suicide than those without children.

Examining the findings regarding the spouse/partner reveals that the suicidal tendencies of women whose spouses work in the public and private sectors are 33.1 and 21.4% less than those whose spouses do not work, respectively. Women whose partners consume alcohol are 23% more likely to commit suicide than women whose partners do not consume alcohol. Women whose spouse or partner fights with another man using physical violence have a suicidal tendency 17.8% higher than other women. The suicidal tendency of women who were cheated on by their spouses/partners is 28.3% higher than those who were not cheated on. The suicidal tendency of women whose spouses/partners prevent them from meeting with friends is 17.4% higher than other women. The suicidal tendency of women who were interfered with by their spouse/partner on how they were dressed was 21.6% higher than those who did not. The suicidal tendency of women whose spouse/partner does not allow them to use social media is 14.8% higher than other women. Finally, women who were exposed to economic, emotional, physical and sexual violence by their spouse/partner were 27.6, 36.3, 33.3 and 49.3% higher, respectively, compared to those who were not.

Suicidal tendencies are 21.5, 54.5, 19.6, and 17% higher in women who have been subjected to physical, sexual, emotional, and economic violence by someone other than their spouse or partner compared to other women. The suicidal tendencies of women whose household income levels are at the first- and third-income levels are 17.9 and 20.8% higher, respectively, than the suicidal tendencies of women whose household income levels are at the fourth or maximum level.

## 4 Discussion

In this study, binary logistic regression analysis was used to examine the factors influencing the suicidal tendencies of married women in Turkey. Many factors affecting the suicidal behavior of women have been identified. In this study, the region, age, education level, health status, number of children, the sector in which the spouse/partner works, the drinking status of the spouse/partner, the spouse/partner’s fight with another man involving physical violence, the spouse/partner’s cheating status, the spouse/partner’s obstruction of friend meetings, the interference with clothing and social media use, the spouse/partner’s economic, emotional, physical and sexual violence, exposure to physical, sexual, emotional and economic violence of someone other than spouse/partner and household income level variables were found to be associated with suicidal tendency of women.

The study found a correlation between the area variable and women’s suicidal behavior. It has been determined that regional employment, differences in regional development, and geographical location can trigger suicidal behavior in women (80). For instance, there is a gradual increase in female suicide from the west to the east of Turkey (81). The region factor can be evaluated both domestically and internationally (80). On a national basis, a positive correlation was found between low- and middle-income countries and suicides (82, 83).

In contrast, a different study found that countries with low and middle incomes have fewer female suicidal tendencies than countries with high incomes (84). It has been observed that female suicide rates vary across continents. The Asian Continent has been found to have the highest female suicide rate (85).

The age of women was found to be connected with suicidal thoughts in the study. Compared to women aged 45–54, it was found that women aged 15–24, 25–34, and 35–44 had greater suicidal tendencies. Similar to these results, in a study conducted in Van province in Turkey between 2005 and 2011, it was found that the majority of women who committed suicide were between the ages of 16 and 20, with statistically significant age differences (86). Another study conducted in Turkey between 1990 and 2010 found that the highest suicide rate was among those aged 15 to 24, and the number of suicides in women was significantly higher than in men (87). A study conducted in Jamaica found that the average age of women who committed suicide was 34 (88). In another study, the increase in suicide rates among women aged 15 to 24 is highlighted (89). In a study comparing the rates of women and men committing suicide, it was found that women who commit suicide are younger than men who commit suicide (90).

One of the most remarkable findings of the study is that women’s suicidal tendencies increase with their level of education. In studies examining the education level of women in suicide attempts and suicide completion behavior, it was determined that individuals who completed suicide were more educated than those who attempted suicide (90). However, in terms of the role of socioeconomic position (education and unemployment) in the relationship between IPV and suicide attempts, a study carried out in the Philippines found that low education level was a risk factor for both intimate partner violence and suicide attempts compared to high or secondary education level (91). In Leon (Nicaragua), it is seen that the education level of women who live in poverty and have a positive attitude toward suicide is at the primary level, and they cannot even complete primary education (92). In a study conducted in Batman province in Turkey, it was determined that those who committed suicide had, on average, 3.9 years of education and that nearly half of the 13 suicide victims were illiterate (93). Similarly, it was determined that the majority of women who applied to Batman Regional State Hospital in Turkey due to suicide attempts were illiterate (70). In another study conducted in the same province, it was determined that the majority of women who committed suicide or attempted suicide were illiterate and had completed primary school (94).

In the study, a correlation between health status and suicidal tendencies was determined. It has been determined that women with excellent or good health are less likely to commit suicide than women with poor or very poor health. Examining the studies reveals that the likelihood of suicidal behavior increases in patients receiving long-term treatment, no treatment, or painful treatment procedures (95–99). Similarly, in a study conducted in the province of Batman in Turkey, it was found that suicidal individuals had a significant mental illness, particularly a severe mental illness such as major depressive disorder (93). According to another study on suicide and suicide attempts in Batman, Turkey, the majority of women committed suicide due to illness (94). According to a study of suicide and suicide attempts in the Muğla region, physical and psychological discomfort are among the causes of both suicide and suicide attempts (100).

The study found a relationship between women’s suicide tendencies and the number of children they had. It has been determined that women with two or more children are less likely to commit suicide than women without children. Similarly, a study examining the relationship between the number of children in a marriage and the suicide rate found that the risk of suicide decreased with increasing numbers of children (101). On the other hand, the study conducted with African-American women revealed that the number of children born to women who attempted suicide and those born to women who did not attempt suicide were comparable (102). In another study in which more than 40% of women attempted suicide, the low percentage of women who had never given birth is notable (92). In another study, it was found that more than 50 % of women with suicidal behaviors had two or three children (103).

The study’s findings indicate that women’s suicide tendencies are correlated with the industry in which the husband or partner works. It has been determined that women whose spouses or partners work in the public or private sector are less likely to commit suicide than those whose spouses are unemployed. Similar to the results of the study, in another study conducted in Iran, it was determined that the officially working spouses of those who attempted suicide had a low suicidal tendency (104). According to another study on intimate partner homicide-suicide, the majority of the perpetrators are unskilled workers (88).

According to the study, there is a correlation between the drinking status of the spouse/partner and the suicidal tendency. In a study, it was stated that there is/may be a relationship between alcohol and suicidal ideation (105). In another study, it was determined that the drinking/gambling habits of a woman’s spouse or partner could trigger suicide attempts (100). According to the study, it was detected that there is a relationship between the spouse/partner fighting with another man in a way that includes physical violence and suicidal tendencies. There are studies in the literature indicating that fighting with a spouse can lead to suicidal thoughts (106, 107).

According to the study, women who experienced controlling behavior from their partners or spouses exhibited higher levels of suicidal thoughts. Similar to the findings of this study, family members of suicidal criminals described them as abusive, jealous, controlling, possessive, and obsessive in a study conducted in Jamaica. In addition, when suicides were examined in depth, a newspaper article revealed that women were not permitted to communicate with men, use social media, or have their phones tampered with (88). Turkey, on the other hand, is characterized by a traditional family structure and a social structure that cannot protect women from violence. The problem that arises from the women’s inability to express their ideas clearly in family relationships, particularly as a result of the pressure they experience, may manifest as a suicide attempt (108). In other studies, it has been found that suicide attempts are very high in women with spouses/partners with controlling behaviors (109, 110).

According to the findings of the study, domestic violence is associated with suicide. Those who experience economic, mental, physical, or sexual abuse at the hands of their spouse or partner are more likely to consider suicide than those who do not. Similarly, it has been determined that African American women who have experienced intimate partner violence and attempted suicide are primarily exposed to physical and non-physical violence (102). Women who have experienced physical, sexual, or any other type of violence are much more likely than those who do not disclose abuse to report mental health issues and suicidal thoughts, according to a study done on women in Delhi (111). A study in Leon (Nicaragua) found that 43.4% of female IPV victims living in poverty attempted to commit suicide at some point in their lives (92). According to research conducted in the Philippines, 8% of the women said that IPV caused them to experience psychological suffering and make suicide attempts (91). A Turkish study found that women who have considered suicide or attempted suicide are more likely to be victims of economic violence (112). Research has indicated that women who witness sexual abuse by their spouses or partners may experience suicidal thoughts (113). On the other hand, in Harare, Zimbabwe, researchers discovered that while sexual violence was not linked to suicidality in women, emotional and physical abuse was (114).

The study found a correlation between exposure to emotional, economic, sexual, and physical abuse by someone other than a spouse/partner and suicidal behavior. Other studies have found that exposure to physical, psychological, and/or sexual abuse by individuals other than a spouse/partner during childhood has a significant impact on the likelihood of committing suicide as an adult (115–117). In another study, it was found that the father’s feelings of argumentation, anger, hostility toward the mother, and emotional violence against the child increased the child’s future suicidal tendencies (118). Another study found a strong correlation between women’s experiences with physical attacks by non-spouses after the age of 18 and their suicide attempts. Additionally, the same study revealed a correlation between sexual abuse experienced after the age of 18 and suicidal ideation (92).

The study discovered a link between women’s suicidal tendencies and household income. As household income decreases, the likelihood of suicidal behavior increases. Consistent with this finding, a Japanese study found that suicide ideation declined with rising household wealth (119). Similarly, a study conducted in South Korea found that when household income decreases significantly, suicidal tendencies increase (120). Additionally, it is stated that suicidal ideation increases in situations where household income decreases, such as retirement (121). In contrast to this study, other research has found that those with a minimum income attempt suicide at a lower rate than those with higher incomes (92, 102).

This research has a number of limitations. First, the study’s data are secondary. The variables required for statistical analysis are already present in the data set. However, some variables, such as the individual’s occupation not in the data set and the homeownership status, could not be analyzed. Second, the data obtained regarding women’s suicidal tendencies consists of the women’s responses. Therefore, the data obtained through this data collection method may be biased. Finally, the data used in the study is from 10 years ago. The most recent National research on domestic violence against women in Turkey data shared by Hacettepe University Institute of Population Studies is that of the year 2014.

## 5 Conclusion

Female suicides are the result of a complex set of factors. This study’s findings are significant for understanding the causes of women’s suicidal behavior and as a source of information on suicide prevention. Even though suicide prevention and women’s support work are crucial to social education and awareness-raising about women’s roles, empowering women in the most deprived environments, and social rejection of violence against women, suicide prevention and support work for women still pose a significant challenge in Turkey. This study’s findings can serve as a valuable guide for the development of culturally appropriate strategies for understanding suicidal behaviors in females for the prevention of suicides. In particular, it can help policymakers and social actors raise the issue’s visibility.

To reduce and prevent women’s suicidal behaviors, more effective results can be achieved by giving priority to women who are particularly between the ages of 15 and 24, reside in the south of Turkey, have a high school diploma, are in poor health, do not have children, have low household income levels, are living with an unemployed spouse/partner, and are exposed to various forms of violence by their partner or non-partner. In addition, effective suicide prevention programs can be created with social policies and mindful practices that will reduce the controlling behaviors of the spouse/partner.

Women can be encouraged to participate more actively in corporate life, penalties can be increased, and facilities can be set up to help women get their emotional lives back on track. It is possible to make sure that women are informed of their legal rights and what to do if they experience one of the forms of violence.

Existing legal protections for women against emotional, economic, sexual, and physical abuse at the hands of individuals other than their spouses/partners can be developed and strengthened. Activities promoting public awareness that are insensitive to the unjust treatment that drives women to suicide are of vital importance.

Suicide as a result of violence against women should be discussed at all levels of education, and awareness campaigns should be organized to raise awareness. Important members of society, including religious and political leaders, artists, and athletes, should be encouraged to participate in the awareness campaign. In addition, instead of sensationalizing the event, media reporters can warn the public about patterns of murder-suicide, dispel myths, and educate the public about responsible reporting.

The regulations envisioned in practice today are inadequate, even though they are meant to prevent and support women who commit suicide. Many violent victims who are in danger of trying suicide think that if they disclose the abuse to the police, they will not be protected and will instead face further violence. To boost public trust in the judicial system, domestic abuse allegations should be handled in police stations and courtrooms openly and understandably. To further prevent the use of firearms for suicide and all other types of violence, it is imperative to support individual disarmament and outlaw the possession of firearms within the home.

## Data availability statement

The data analyzed in this study is subject to the following licenses/restrictions: the data underlying this study is subject to third-party restrictions by the Turkey Statistical Institute. Data are available from the Turkish Statistical Institute (bilgi@tuik.gov.tr) for researchers who meet the criteria for access to confidential data. The authors of the study did not receive any special privileges in accessing the data. Requests to access these datasets should be directed to bilgi@tuik.gov.tr.

## Author contributions

ŞK: Conceptualization, Data curation, Formal analysis, Methodology, Supervision, Writing – original draft, Writing – review & editing. SÇ: Data curation, Formal analysis, Methodology, Writing – original draft, Writing – review & editing. AG: Conceptualization, Writing – review & editing. ÖA: Conceptualization, Data curation, Formal analysis, Methodology, Writing – original draft, Writing – review & editing.
